# Dysregulation of Cell Death and Its Epigenetic Mechanisms in Systemic Lupus Erythematosus

**DOI:** 10.3390/molecules22010030

**Published:** 2016-12-27

**Authors:** Haijing Wu, Siqi Fu, Ming Zhao, Liwei Lu, Qianjin Lu

**Affiliations:** 1 Department of Dermatology, Second Xiangya Hospital, Central South University, Hunan Key Laboratory of Medical Epigenomics, Changsha 410011, China; chriswu1010@126.com (H.W.); 18874139898@163.com (S.F.); zhaoming307@126.com (M.Z.); 2 Department of Pathology and Center for Infection and Immunology, the University of Hong Kong, Hong Kong, China

**Keywords:** SLE, cell death, apoptosis, necrosis, autophagy, epigenetics

## Abstract

Systemic lupus erythematosus (SLE) is a systemic autoimmune disease involving multiple organs and tissues, which is characterized by the presence of excessive anti-nuclear autoantibodies. The pathogenesis of SLE has been intensively studied but remains far from clear. Increasing evidence has shown that the genetic susceptibilities and environmental factors-induced abnormalities in immune cells, dysregulation of apoptosis, and defects in the clearance of apoptotic materials contribute to the development of SLE. As the main source of auto-antigens, aberrant cell death may play a critical role in the pathogenesis of SLE. In this review, we summarize up-to-date research progress on different levels of cell death—including increasing rate of apoptosis, necrosis, autophagy and defects in clearance of dying cells—and discuss the possible underlying mechanisms, especially epigenetic modifications, which may provide new insight in the potential development of therapeutic strategies for SLE.

## 1. Introduction

Systemic lupus erythematosus (SLE) is a systemic autoimmune disorder that predominantly affects women (the female to male ratio is 9 to 1) [1]. It is characterized by an abundance of autoantibodies in the circulation of patients [2], together with aberrant innate and adaptive immune responses [3,4]. Although the exact etiology of SLE remains unclear, many factors have been shown to play a role in autoimmune pathogenesis of SLE, including genetic susceptibility and various environmental factors [5]. SLE occurs when an individual with genetic susceptibility encounters environmental triggers such as sunlight, drugs, or infection. The majority of these triggers may induce cell death in an epigenetically-regulated fashion and lead to increased exposure, availability, and immunogenic characteristics of intracellular self-antigens. Consequently, immune tolerance is broken down which may result from defects in clearance of dead cell materials and abnormal presentation of auto-antigens by antigen-presenting cells (APCs) to T cells. Upon activation by self-antigens, T cells provide help to auto-reactive B cells, leading to production of abundant auto-antibodies. As a result, these auto-antibodies bind to self-antigens by forming immune complexes (ICs) that are deposited in multiple organs, leading to organ inflammation, dysfunction, and failure [6,7,8,9]. In this complex disease process, abnormal cell death may act as an initial trigger for the development of autoimmune disorders.

### 1.1. Dysregulated Apoptosis in SLE

Apoptosis, also called programmed cell death, is a main cellular mechanism for body to maintain tissue homeostasis. Apoptosis is induced by activation of extrinsic pathway involving membrane receptors such as Fas or intrinsic pathway involving Bcl-2 family proteins [10]. Factors that trigger apoptosis include intracellular p53 signaling and extracellular factors such as chemotherapy, radiation, cell stress, and inflammation. All stimuli above lead to activation of capase-3 that initiates the process of apoptosis. Dysregulation of apoptosis are linked to the pathogenesis of SLE since cellular debris from apoptotic cells are the likely sources of auto-antigens in SLE. Increased apoptosis of immune cells, particularly T lymphocytes, has been reported in PBMCs and cutaneous lesions from SLE patients [11,12,13,14]. Serum samples from SLE patients were found to induce apoptosis among normal lymphocytes and neutrophils [15,16], which may be related to increased levels of soluble Fas ligand (FasL) in lupus serum activating Fas/FasL via the extrinsic pathway of apoptosis [16,17]. In addition, exposure to UV light, a potent inducer for apoptosis [18], has a strong association with lupus development [19].

**Figure 1:**
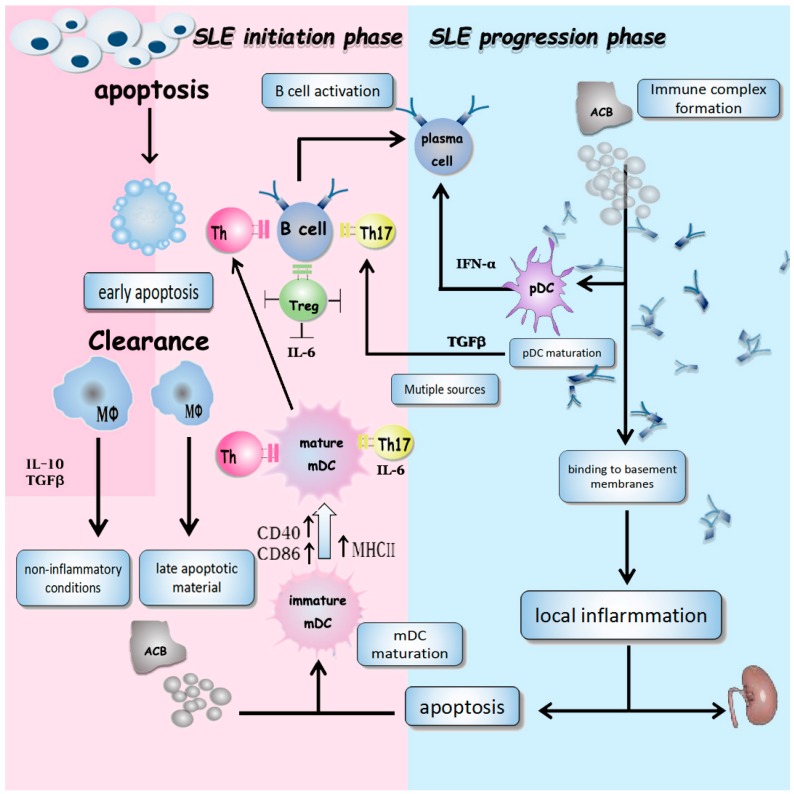
The role of apoptosis in the development of SLE. At the initiation phase of SLE, macrophages have defects in cleaning up apoptotic cells. The cellular materials from apoptotic cells can be taken up by immature DCs (imDCs). The imDCs therefore undergo a maturation stage and become mature DCs with the upregulated CD40, CD86, and MHC-II levels. Mature DCs then polarize Th0 cells into Th1, Th2, and Th17 cells, which help B cell differentiation and activation. At the same time, apoptotic bodies bind to antibodies and form immune complexes (ICs). ICs further activate pDCs and promote the production of IFN-α, which promotes antibody production and isotype switching. ICs are also deposited in renal tissue, leading to immune cell infiltration and local tissue damage, which can induce more apoptosis, forming a positive loop in the progression phase of SLE. ACB, apoptotic cell body (“late apoptotic cell”); Blebs, apoptotic blebs; EAC, early apoptotic cell; MF, macrophage; mDC, myeloid dendritic cell; Nucleosomes, apoptosis-induced hyper acetylated nucleosomes; pDC, plasmacytoid dendritic cell; Th, T helper cell; Treg, regulatory T cell.

However, how exactly apoptosis contributes to SLE remains partially understood. In SLE, apoptotic blebs and nucleosomes are formed during apoptosis and become the source of auto-antigens. These cellular materials are taken up by immature myeloid dendritic cells (imDCs) undergoing maturation by expressing high levels of costimulatory molecules (CD86, CD40, and MHC-II) and pro-inflammatory cytokines, including IL-6 and IL-12p70. Mature DCs activate T helper 1 cells (Th1) and Th2 cells through CD86 and CD40 binding to their ligands. Moreover, mature DCs can also polarize naïve T cells (Th0) to Th1 cells under influence of IL-12p70. Under the effects of IL-6, regulatory T cells are inhibited whereas Th17 cell differentiation is promoted. With help from T cells, autoreactive B cells produce autoantibodies against self-antigens. Subsequently, autoantibodies are involved in forming immune-complexes (ICs) after binding to auto-antigens. In addition, after taking up ICs containing nucleic acids, plasmacytoid dendritic cells (pDCs) produce high amount of IFN-α, which then promote antibody production and isotype switching. ICs are deposited in renal tissue, resulting in immune cell infiltration and local tissue damage, which can further induce more apoptosis (Figure 1) [20] acting as a positive loop to break down immune tolerance and accelerate disease progression.

### 1.2. Epigenetic Modification-Mediated Increased Apoptosis in SLE

Epigenetics, the study of the reversible and potentially heritable changes in gene expression without genetic code alterations—including DNA methylation, histone modification, and microRNAs (miRNAs)-mediated regulation—is a new area of investigation into the pathogenesis of SLE [21]. Lines of evidence have shown the involvement of epigenetic alterations such as miRNAs-mediated regulation and DNA methylation in the process of apoptosis.

#### 1.2.1. miRNAs-Mediated Apoptosis in SLE

Recent studies have demonstrated that miRNAs are small, non-coding RNAs (21–25 base pairs) and function as posttranscriptional and posttranslational regulators of gene expression. miRNAs exert their functions by binding to the 3′-untranslated region (UTR) of the messenger RNA (mRNA) of a target gene, causing mRNA cleavage, translational repression, or translational arrest [22,23], thereby silencing the gene expression. Similar to the increased rate of apoptosis, decreased apoptotic rate of T cells may also contribute to SLE. The lupus-prone mouse model (MRL/lpr) with a mutation on Fas displays a defective apoptosis, a typical example of over-proliferating T and B cells. This results in lupus-like cutaneous lesion and nephritis. Indeed, several anti-apoptosis microRNAs are found to be decreased in lupus patients while the pro-apoptotic microRNAs are increased in the circulation of SLE. miR-17-92 family is capable of inhibiting apoptosis by suppressing the Bim and PTEN expression [24], which shows reduced levels in lupus sera [25]. Interestingly, increased levels of miR-29b and miR-29c, targeting anti-apoptotic member Bcl-2 have been observed in lupus pDCs after the administration of glucocorticoids [26], as well as in lupus T cells [27]. In addition, miR-21 can suppress apoptosis of activated T cells by targeting tumor suppressor gene Tipe2 [28], which is also increased in lupus patients [29,30].

#### 1.2.2. DNA Methylation and Apoptosis

DNA methylation is a biochemical process in which a methyl group is added to a cytosine or DNA methylation is a biochemical process in which a methyl group is added to a cytosine or adenine at the 5′ position of a CpG dinucleotide, converting the cytosine to methylcytosine [31]. In general, CpG island methylation is associated with gene silencing. Methylation serves as a mark that indicates repression of gene expression. Therefore, DNA methylation is involved in many biological processes such as cell differentiations, apoptosis, and immune regulation. Although there is lack of direct evidence on DNA methylation-mediated apoptosis in SLE, abnormal expression of DNA methylation-mediated genes involved in regulating apoptosis have been reported to be aberrantly expressed in SLE patients. For example, p53, a tumor suppressor gene with the capacity of inducing apoptosis by altering the expression of genes with p53 binding sites, is found to be downregulated by aberrant methylation in tumor cells [32,33]. On the contrary, increased p53 protein expression and cell apoptosis rate has been observed in discoid lupus skin lesion [34] and peripheral blood [35]. Moreover, DNA demethylation has been detected in CD4^+^ T cells, but not in CD8^+^ T cells or peripheral blood mononuclear cells (PBMCs) from SLE patients [36,37]. This evidence may suggest that DNA hypomethylation of the p53 gene contributes to the enhanced p53 expression and causes abnormal apoptosis in lupus patients, a notion that needs further investigation.

Several studies have demonstrated that methyl-CpG-binding domain 4 (MBD4) can be recruited by methylated DNA and thereby leading to the recruitment of histone modifying and chromatin-remodeling complexes to methylated sites [38,39]. MBD4 is involved in the apoptosis signaling pathway [40] and found to be overexpressed by lupus CD4^+^ T cells [41]. Furthermore, Fas-FasL [42] and PD-1-PD-L1 [43] pathways are linked with lupus pathogenesis [13,44,45], which are also regulated by DNA methylation and participated in the apoptosis process. Both PD-1 and PD-L1 molecules are overexpressed by immune cells and cancer cells respectively due to the loss of 5-hmc [46] or hypomethylation [43] on the promoter region, which may provide potential therapeutic targets for autoimmune disorders and cancers.

### 1.3. Impaired Clearance of Apoptotic Materials in SLE

**Figure 2:**
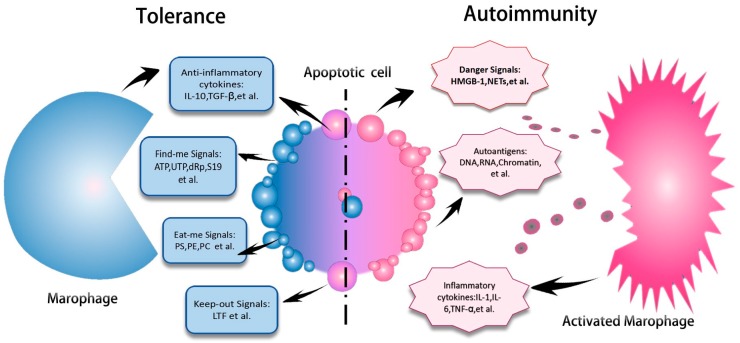
Apoptotic cells induce immune tolerance and autoimmunity. Early apoptotic cells secrete anti-inflammatory cytokines—such as IL-10, TGF-beta—and express find-me (ATP, UTP, dRp S19), eat-me (PS, PE, PC), and keep-out (LTF) signals to macrophage for tolerance induction. However, when the clearance of early apoptotic cells is impaired, they will undergo late apoptotic process and release danger signals such as HMGB-1 and NETs as well as exposure auto-antigens (DNA, RNA, and chromatin). Macrophages are activated by these danger signals and then produce inflammatory cytokines—including IL-1, IL-6, and TNF-α—which may contribute to the development of autoimmune disorders. ATP, adenosine triphosphate; UTP, uridine triphosphate; dRp S19, dimer of ribosomal protein S19; PS, phosphatidylserine, PC, phosphatidylcholine; PE, phosphatidylethanolamine; LTF, Lactotransferrin.

Under homeostasis, apoptotic cells are rapidly cleared by resident macrophages from tissues. Otherwise, accumulated apoptotic cells may induce either inflammation or immune responses leading to the breakdown of immune tolerance and autoimmune pathogenesis. Early apoptotic cells secrete anti-inflammatory cytokines, such as IL-10, TGF-beta, and express find-me (adenosine triphosphate (ATP), uridine triphosphate (UTP), dimer of ribosomal protein S19 (dRp S19)), eat-me (phosphatidylserine (PS), phosphatidylcholine (PC), phosphatidylethanolamine (PE)), and keep-out (Lactotransferrin (LTF)) signals to macrophages for tolerance induction [47]. Macrophages that have ingested apoptotic cells secrete ‘tolerate me’ cytokines, including transforming growth factor β (TGFβ) and IL-10, which create an anti-inflammatory milieu at the sites of cell death (Figure 2). Early studies have observed impaired phagocytic capacity of monocyte-derived macrophages from SLE patients [48,49,50,51]. Moreover, defective phagocytosis is also linked to decreased C reactive protein level and complement consumption in the sera of lupus patients [52].

When the clearance of apoptotic cells is impaired, excessive apoptotic cells undergo secondary necrosis and release danger signals to break down the immune tolerance. Danger signals including nucleosomes, non-histone nuclear proteins, metabolic intermediates, and nuclear antigens can activate the NF-κB pathway and inflammasomes to produce pro-inflammatory cytokines and promote survival of autoreactive lymphocytes [53]. Recently, elevated levels of the high mobility group box 1 (HMGB1) have been detected in the sera of SLE patients [54,55]. In addition, the secondary necrotic cells (SNECs) can promote the production of auto-antibodies [56], while SNEC-containing ICs can stimulate pathogen recognize receptors (PRRs), leading to the production of IL-6, IL1β, IL-8, and abnormal activation of DCs [52], contributing to the development of SLE.

#### Epigenetic Mechanisms Underlying Impaired Phagocytic Function of Macrophages

Currently, there is little evidence revealing the link between DNA methylation and macrophage functions. However, it has been recently reported that DNA demethylation is detected in the sphingosine-1 phosphate receptor 5 (S1PR5), which may explain the observed defects in S1P system and phagocytic ability in macrophages [57]. Notably, an increased sera level of S1P has been found in juvenile-onset SLE [58], suggesting a link between DNA methylation-mediated macrophage function and the pathogenesis of SLE. It has become clear that both differentiation and maturation of macrophages are indeed regulated by epigenetic modifications [59]. However, further studies are needed to establish the link among epigenetics, macrophages, and SLE.

### 1.4. NETosis and SLE

Neutrophil extracellular traps (NETs) act as an important defense mechanism during infection by trapping and killing bacteria via the extrusion of nuclear material including DNA and histones in a web-like structure [60]. NETs are composed of histones, chromatin, and proteins secreted by granules—including elastases, myeloperoxidases, matrixmetalloproteinases 9, pentraxin 3 and antimicrobial molecules such as cathelcidins [61,62]. NETs are mainly released by activated neutrophils that undergo a novel cell-death mechanism named NETosis [63]. Under normal conditions, extracellular NETs can limit inflammation through degrading chemokines and cytokines [64]. However, NETs can also become a harmful source of auto-antigens and contribute to autoimmune diseases such as SLE [64]. When NETs are not promptly cleared, they expose dsDNA molecules and aggregate decorated with endogenous and exogenous immunostimulatory molecules, prone to promote auto-antibody production [65]. NETs also activate caspase-1, a key enzyme in inflammasome, thereby promoting the release of an active form of IL-1beta and IL-18 [66], which consequently drives Th17 differentiation and promotes autoimmune inflammation. In addition, most of the antigens released by NETs appear to become more immunogenic through post-translational modifications. For example, cirtullinated histones are preferentially recognized by antibodies in lupus patients [67]. In fact, most specific auto-antigens in SLE are NET components, indicating a role of excessive NETosis and defective clearance of NETs in SLE [68]. Increasing evidence has revealed the presence of excessive NETs in the inflammatory sites of lupus including glomeruli [69] and skin [64]. In lupus patients, both IFN-α and immune complexes, are potential triggers of NETosis [67]. Recently, the reduced clearance of NETs is also observed in lupus patients [70], which may result from the presence of D-Nase1 inhibitors or anti-NET antibodies. Since mere exposure to NETs may not result in the development of autoimmune diseases, additional triggers such as infection need to be identified [71].

### 1.5. Autophagy and SLE

Autophagy is a lysosome-mediated catabolic process to maintain intracellular homeostasis via disposal of damaged, non-functional, or unnecessary proteins and organelles. Autophagy is also involved in immune regulation and plays a critical role in the development, survival, and proliferation of T and B lymphocytes [72,73,74]. Autophagy involves three morphological phases: initiation (i.e., formation of phagophores), elongation, and closure (i.e., increase in the size of the phagophore and its closure into a completed autophagosome), and maturation (i.e., conversion of autophagosomes into degradative organelles, termed autophagolysosomes, by fusion with late endosomal and lysosomal organelles). The serine/threonine kinase mammalian target of rapamycin (mTOR) plays a key role in the regulation of autophagy. DNA-ICs are phagocytized by pDCs, inducing IFN-α production via toll like receptor 9 (TLR9) activation, which requires a noncanonical autophagy pathway called LC3-associated phagocytosis (LAP). LC3 amplifies the response to intracellular DNA, and another important member of the autophagy pathway, Beclin-1, serves as an inhibitor, thereby suppressing IFN-α production, a key effector cytokine in lupus pathogenesis. The perturbation of autophagy has been linked with autoimmune disorders, especially SLE. The first indirect evidence was reported in 1964, which demonstrated the association of lysosomal function and SLE [75]. In recent studies, a strong connection between autophagy and lupus has been revealed by the findings of single-nucleotide polymorphisms (SNPs) in autophagy-related gene 5 (Atg5) to lupus susceptibility [76,77,78], which is further supported by the findings of an important role of Atg7 in B cell development and activation of autophagy in SLE patients [79]. It has been reported that decreased levels of Beclin-1, mTOR, and LC3-II are detected in lupus patients [80]. Interestingly, currently used drugs for SLE treatment can regulate autophagy. Dexamethasone has been shown to induce autophagy via inhibiting mTOR phosphorylation [81]. Moreover, the commonly used hydroxychloroquine exhibits therapeutic effects by lysosomal acidification [82]. In addition, vitamin D also induces autophagy via interfering lysosomal acidification, thereby inhibiting the degradation of autophagic vacuoles [83]. Furthermore, cyclosporine A and rapamycin have been found to suppress mTOR and inhibit autophagy [80]. Therefore, further investigations are needed to elucidate the role of autophagy in the pathogenesis and treatment of SLE.

Although there is evidence that autophagy is regulated by epigenetics, our current understanding of SLE etiopathogenesis is still very limited. For histone modification, histones present small protein tails protruding from the nucleosome, which is accessible to modifications including methylation, acetylation, and ubiquitination [84]. It has been shown that each modification has a specific function. For example, histone H3K9 acetylation enhances transcription whereas methylation shows an opposite effect. It is well known that the addition of H3K4me3 enhances gene expression, whereas H3K9me3 and H3K27me3 lead to gene downregulation [85,86]. Sirtuin-1 (Sirt-1), a histone deacetylase, has been shown to regulate autophagy by increasing mitochondrial function and reducing oxidative stress [87]. The elevated expression level of Sirt-1 has been reported in lupus-prone MRL/lpr mice [88]. Furthermore, the downregulation of Sirt-1 results in short-term enhanced H3 and H4 acetylation, with reduced levels of anti-dsDNA, glomerular IgG deposition, and kidney damage [36]. 

### 1.6. Necroptosis and SLE

Necroptosis is another important form of cell death via a caspase-independent defense mechanism against intracellular pathogens, which is triggered by activation of TNF and Fas-FasL pathways. Recent studies have shown that IFN-α has the capacity of necroptosis induction in a mouse model [89]. IFN-α receptor-deficient macrophages are resistant to Salmonella-induced necroptosis [90], suggesting a possible role of necroptosis in the IFN-α abundant lupus patients. Indeed, increased necroptosis has been observed in B cells from active lupus patients [91]. To date, there are few studies on necroptosis in SLE, but extensive studies on TNF-like weak inducer of apoptosis (TWEAK) have indicated a central role for the TNF pathway in the induction of lupus nephritis [92].

## 2. Conclusions

Current studies have shown that dysregulated cell death contributes to SLE by increasing availability, pathogenic modifications, and abnormal processing and presenting of self-antigens. Epigenetic modifications involve this complex process at different levels, including triggering apoptosis at the initial phase, induction of impaired clearance of apoptotic materials by inappropriate DNA methylation, and aberrant antigen presentation or IFN-α production by DCs [93]. To induce the apoptosis of over-activated immune cells, recombinant PD-L1Ig has been proposed as a therapy in lupus [94]. Nevertheless, further studies on epigenetics-mediated apoptosis and its role in SLE development are needed to deepen our understanding of etiopathogenesis, which may facilitate the development of novel therapeutic strategies for treating autoimmune diseases.
